# Using a State-Space Model and Location Analysis to Infer Time-Delayed Regulatory Networks

**DOI:** 10.1155/2009/484601

**Published:** 2009-09-07

**Authors:** Chushin Koh, Fang-Xiang Wu, Gopalan Selvaraj, Anthony J Kusalik

**Affiliations:** 1 Department of Computer Science, University of Saskatchewan, Saskatoon, SK, Canada, S7N 5C9; 2 Department of Mechanical Engineering, University of Saskatchewan, Saskatoon, SK, Canada, S7N 5A9; 3 Division of Biomedical Engineering, University of Saskatchewan, Saskatoon, SK, Canada, S7N 5A9; 4 Plant Biotechnology Institute, National Research Council of Canada, Saskatoon, SK, Canada, S7N 0W9

## Abstract

Computational gene regulation models provide a means for scientists to draw biological inferences from time-course gene expression data. Based on the state-space approach, we developed a new modeling tool for inferring gene regulatory networks, called time-delayed Gene Regulatory Networks (tdGRNs). tdGRN takes time-delayed regulatory relationships into consideration when developing the model. In addition, a priori biological knowledge from genome-wide location analysis is incorporated into the structure of the gene regulatory network. tdGRN is evaluated on both an artificial dataset and a published gene expression data set. It not only determines regulatory relationships that are known to exist but also uncovers potential new ones. The results indicate that the proposed tool is effective in inferring gene regulatory relationships with time delay. tdGRN is complementary to existing methods for inferring gene regulatory networks. The novel part of the proposed tool is that it is able to infer time-delayed regulatory relationships.

## 1. Introduction

Microarray technology allows researchers to study expression profiles of thousands of genes simultaneously. One of the ultimate goals for measuring expression data is to reverse engineer the internal structure and function of a transcriptional regulation network that governs, for example, the development of an organism, or the response of the organism to the changes in the external environment. Some of these investigations also entail measurement of gene expression over a time course after perturbing the organism. This is usually achieved by measuring changes in gene expression levels over time in response to an initial stimulation such as environmental pressure or drug addition. The data collected from time-course experiments are subjected to cluster analysis to identify patterns of expression triggered by the perturbation [1,2]. A fundamental assumption is that genes sharing similar expression patterns are commonly regulated, and that the genes are involved in related biological functions. Biologists refer to this as "guilt by association." Some frequently used clustering methods for finding coregulated genes are hierarchical clustering, trajectory clustering, -means clustering, principal component analysis (PCA), and self-organizing maps (SOMs). A general review of these clustering techniques is presented by Belacel et al. [3]. 

A gene network derived by the above clustering methods is often represented as a wiring diagram. Cluster analysis groups genes with similar time-based expression patterns (i.e., trajectories) and infers shared regulatory control of the genes. The clustering result allows one to find the part-to-part correspondences between genes. The extents of gene-gene interactions are captured by heuristic distances generated by the analysis. The network diagram produced provides insights into the underlying molecular interaction network structure.

Two major limitations of conventional clustering methods are that  they cannot capture the effects of regulatory genes that are not included in the microarray;  they do not account for transcriptional time delay which occurs in cells. For example, transcription of a gene depends on the assembly of a transcribing complex, and that complex typically contains several proteins. Some of these are core proteins that catalyze mRNA synthesis and others are factors that modulate mRNA synthesis according to the genetic and environmental specifications for a given gene. Consequently, transcription of such genes is delayed due to the time needed for the production and assembly of the corresponding transcription factors and their assembly into a transcription-competent complex. An example of this is p53 and mdm2 as discussed by Bar-Or et al. [4] where over-expression of p53 triggers a negative feedback mechanism. First, p53 stimulates expression of the mdm2 gene. The production of mdm2 protein in turn represses the transcriptional functions of p53 and promotes p53 proteolytic degradation [5]. Under stress conditions, p53 and mdm2 proteins undergo damped oscillations where mdm2 peaks with a delay of about 60 minutes relative to p53 [4]. In another example Ota et al. [6] conducted a comprehensive analysis of delay in transcriptional regulation using gene expression profiles in yeast.

Wu et al. [7] propose the state-space approach to model gene regulatory networks. Their research results have shown that a state-space model can grasp a number of properties of real-life gene regulatory networks. Recently, Hu et al. [8] compared state-space models, fuzzy logical models, and Baysian network models for gene regulatory networks. Rangel et al. [9,10] apply state-space modeling to -cell activation data. The technique provides a means for constructing reliable gene regulatory networks based on bootstrap statistical analysis. The method is applied to highly replicated data. The confidence intervals of gene-gene interaction matrix elements are estimated by resampling with replacement as many as 200 times. This approach, however, has a severe limitation for application to microarray data because most currently available time-course microarray data are either replicated over only a few time points () or not replicated at all.

The above state-space models [7–10] do not take time delay in gene regulatory networks into consideration. However, examination of microarray data reveals a considerable number of time delayed interactions, suggesting that time delay is ubiquitous in gene regulation [11]. From a biological viewpoint, time delay in gene regulation arises from the delays characterizing the various underlying processes such as transcription, translation, and transport. For example, time delays in regulation may stem from the time taken for the transport of a regulatory protein to its site of action.

Recently, state-space models with time delays have been proposed to account for the effects of missing data and complex time delay relationships. In earlier work we developed a state-space model with time delay to model yeast cell-cycle data [12], and the model was demonstrated on nonreplicated data. Our previous method [12] emphasized identification of a set of internal state variables that govern the cell-cycle process. It assumed that one gene does not directly regulate another and thus does not partition the data set. The drawbacks of this technique are that it is not clear how a network can be derived from the modeling tool, and it is hard to validate the model against biological knowledge of time delay effects. In the same vein, Sung et al. [13] presented a discretized Bayesian network model to construct a multiple time delay gene network using the same data set. The Sung et al. method focused on finding regulatory relationships and associating the regulatory time delay with every "parent-child" (i.e., regulator-target) pair [13]. The data set was partitioned into parent set (the regulators) and child set (the targets). The method suggested a new network structure learning algorithm, Learning By Modification (LBM), to identify potential regulators and then associate them with target genes. 

These existing state-space modeling techniques do not incorporate the structure of gene regulatory networks derived from biological knowledge. Alternatively, Li et al. [14] have published their work on inferring transcription factor activities using a discretized state-space modeling technique. The Li et al. approach incorporates the results of ChIP-on-chip (genome wide location analysis) experiments into the model building. The network structure is predetermined on the basis of a given transcription factor binding to various gene probes in chromatin immuno-precipitation (ChIP)-on-chip assays. The transcription factor activities are then inferred with mathematical modeling using time-course experiments. However, the Li et al. technique does not take time delay into account.

To complement these existing methods, we have developed a new modeling tool called tdGRN for inferring time delayed gene regulatory networks. tdGRN generates a state space-based model into which time delays and the ChIP-on-chip data are incorporated to infer a biologically more meaningful network. A more extensive treatment of tdGRN and the use of state-space modelling with time-series microarray data can be found in the thesis of Koh [15].

## 2. Methodology

The tdGRN approach consists of three parts. First, we implement a state-space model which incorporates multiple time delays. Secondly, we incorporate ChIP-on-chip data for determining network connectivity for both nonreplicated and replicated data. This involves replacing Rangel's bootstrap confidence intervals (derived from highly replicated data) for identifying gene-gene interaction with a substitute. Finally, the networks generated from the new model are visualized using techniques from the literature [16].

### 2.1. Time Delay Model

**Figure 1:**
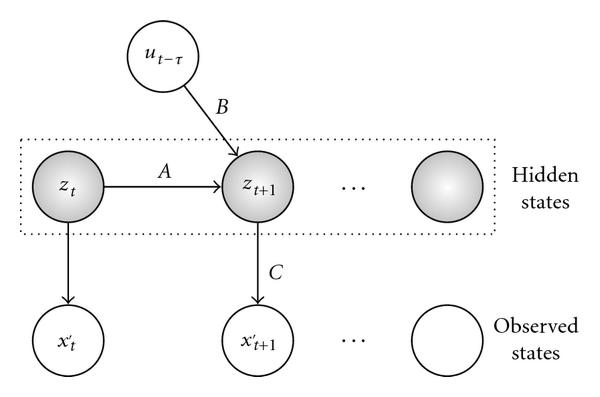
Bayesian network representation of the new model for gene expression.

We consider the expression profile of a regulator (e.g., a transcription factor) as an input function to the system. Therefore, the time period, , from the over-expression of the regulator to the over- or under-expression of the targeted gene is represented as an input-delay function. A gene regulatory system with  regulators,  target genes, and  state variables can be described using the following state-space model with time delays: (1)

where  is an  state transition matrix.  is an  input matrix which captures the impacts of the expression of  regulators on the system.  is a  output matrix that represents the influence of internal state variables on the output gene expression level at each time point.  is an -dimensional vector collecting the values of  state variables at time point .  is a-dimensional vector collecting expression values of  genes at time point .  is a *p-*dimensional vector collecting the values of  input variables at delayed time point .  and  are independent white noises. Compared to the Rangel model, our model removes the feed-forward matrix, , assuming that gene-gene regulation can be captured by indirect regulations through internal variables instead of direct gene regulation from one time point to the next. As with the model by Rangel et al. [10], the product  produces a  matrix that depicts the regulatory relationships between  regulators and  target genes. The possible values for the time delay for each of the  regulators*,*, where , is estimated by scanning a range of positive integers, with the minimum time delay of zero, that is, gene coregulation. The best fit is determined by minimizing the Akaike's Information Criterion (AIC) for the residual variance. AIC was developed by Akaike [17] to determine a compromise between the complexity of an estimated model and the fitness of the model with the data in order to avoid the overfitting problem. A Bayesian network representation of the model is shown in Figure 1. From the results in [12,18], such a modeling approach can assure that the inferred networks are stable and controllable.

The model was implemented as a MATLAB program. tdGRN uses various functions from MATLAB's Control System and System Identification toolboxes. The n4sid() and aic() functions are used for system identification, system stability, and delay analysis. The n4sid() function implements the Numerical Algorithms for State Space Subspace System Identification (NS4SID) proposed by Van Overschee and De Moor [19]. It computes the parameterization of the model, solving for the matrices , , and . The subspace algorithm is noniterative and does not depend on a priori parameterization. This allows the method to always find a convergent system and avoids problems such as local minima and initial condition bias. The system identification is based on QR and singular value decomposition which ensures that the estimated linear time-invariant model is stable [19]. The only requirement for the identification is the order of the system. In tdGRN, the order is determined by selecting the model that produces the best AIC score [12] as computed by the aic() function. The lower the AIC score the better the goodness-of-fit of the estimated state-space model. Finally, the compare() function is used to determine the overall model fitness to the data. The model fitness is represented as a percentage estimated as follows: (2)

where  is the actual gene expression profile, is the mean of , and  is the predicted expression profile from the model.  is the total number of time points.  and  are the Euclidean distances between the predicted and the actual expression profiles, and between the actual expression profiles and mean expression profile, respectively. Ideally, if the distance between the predicted and the actual expression profiles is zero, the function returns a 100% fitness. tdGRN supports two types of models: single input and multiple input models, both with time delays. A single-input model captures simple one-to-one regulatory relationships. A multiple-input model works for complex many-to-one regulatory relations.

### 2.2. Single-Input Model with Delay

**Figure 2:**
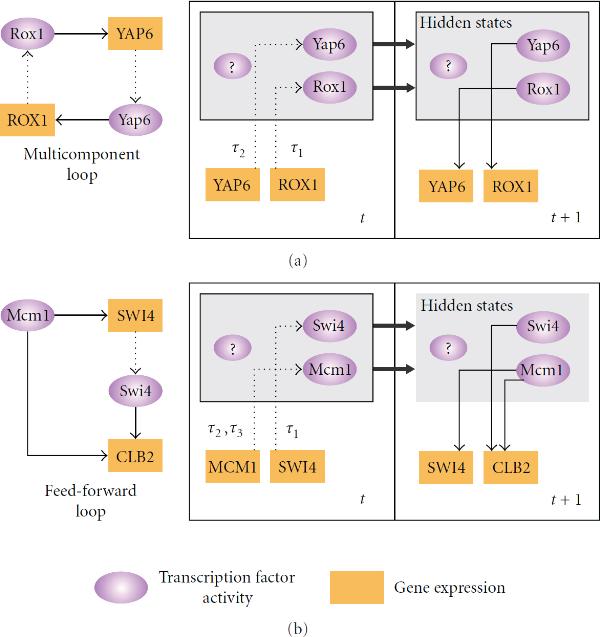
**An example of SISO state-space representation of the gene regulatory network motifs described by Li et al. **[14]. (a) Multicomponent loop, and (b) feed-forward loop. The network motifs are shown on the left and the corresponding state-space models on the right. Purple ellipses correspond to protein expression, while the orange rectangles signify gene expression. All uppercase names are used for transcripts, and mixed upper-and lowercase is used for transcription factor names. A directed dashed line shows the direction of translation, while a directed solid line represents the direction of transcription regulation.

In a simple one-to-one regulatory relation, the regulation of a gene is highly related to its transcription factor (TF). In other words, residual regulation by other factors can be treated as hidden variables, that is, missing data. Therefore, a single-input and single-output (SISO) model (TF versus gene or TF versus TF) can be used to describe the input and output signals. The SISO model can be applied to identify network motifs such as feed-forward loops, Multi-component loops, and single-input motifs as described by Lee et al. [20]. Figure 2 illustrates how tdGRN is used to model two such network motifs. The network motifs are shown on the left and the corresponding state-space models on the right. 

According to Lee et al. [20], two anaerobic condition-related transcription factors in yeast, Rox1 and Yap6, form a regulatory circuit in which they regulate each other. The regulation circuit is represented as a multi-component loop motif as shown in Figure 2(a), where the over- or under-expression of one TF regulates the gene expression of another (i.e., ). In the state-space representation of tdGRN, the mRNA expression of ROX1 and YAP6 (orange boxes) over time are the observed values. The TF protein expression levels, Rox1 and Yap6 (purple ellipses), and possibly other hidden factors (purple ellipse labelled with a question mark, "?") are the hidden variables. At time , the protein expression levels are affected by gene expression of ROX1 and YAP6 with  and  input time delays, respectively. The hidden variables in turn dictate the output gene expressions of ROX1 and YAP6 at time . The multiple time delay relationships can be expressed as a  matrix as follows:(3)

Recall that this  matrix captures the regulatory relationship between the  regulators and the  target genes.

Another example of a network motif is the regulation of CLB2, a G2/M-cyclin gene, and transcription factor Swi4 by Mcm1. It is illustrated by Lee et al. [20] as an example of a feed-forward motif. The MCM1 gene regulates CLB2 as well as the Swi4 transcription factor, which also regulates CLB2 cyclin. In this network motif, there are two regulators, two target genes (i.e., ), and three possible input time delays, each corresponding to a regulatory relation (refer to Figure 2(b)). The multiple time delay relationships are expressed as a  matrix as follows:(4)

The time delay, , is estimated by scanning a range of possible integers, with the minimum time delay of zero, that is, gene coregulation. In the case of yeast cell cycle data, the maximum number of delays should not exceed the time for a complete cell cycle (G1→S→G2→M), which is estimated to be about 60 minutes [13]. For Spellman's time-course microarray data [21], since each sampling interval is 7 minutes, the maximum delay should never exceed 8 sampling intervals (i.e., 60 minutes1 sample/7 minutes). Similar to Li et al. [14] but unlike Ota et al. [6] and Sung et al. [13], we believe that the actual time delay between binding and transcription is on the order of minutes. This is based on an assumption that gene transcriptional regulations are most likely to occur within the same phase or at the transition point from one phase to another. Since the longest cell-cycle phase, G1, takes about 25 minutes, the maximal reasonable delay is less than 3 sampling intervals (i.e., 25 minutes1 sample/7 minutes). Hence, the default maximal delay for yeast cell cycle is set at 2 sampling intervals, that is, 14 minutes, for Spellman's data [21]. Note that this default value may not be applicable to other biological systems.

### 2.3. Multiple-Input Delay Model

A SISO model may not work well when multiple regulators show significant regulation of a target gene. The presence of two or more regulators increases the model complexity. In addition, some studies have shown that different gene pairs have different time delays for gene regulation [13]. Therefore, the multiple time delay issue should also be addressed. We present a multiple-input model with time delay in which the transcription profiles of all known regulators, if available, are provided as inputs to the system. The input delays are estimated individually for each regulator. The multiple-input single-output (MISO) model can be used to determine multi-input and regulator cascade network motifs, as described by Lee et al. [20].

**Figure 3:**
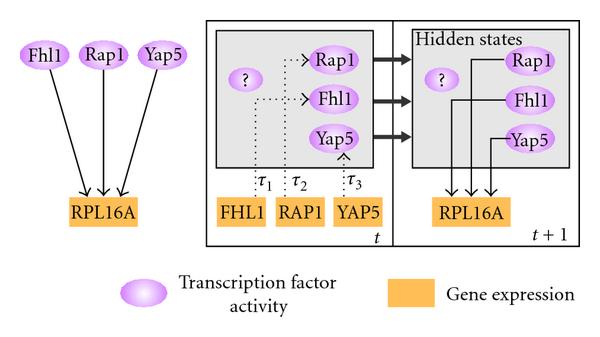
**An example of MISO state-space representation of a multi-input gene regulatory network motif described by Li et al. **[14]. The network motif is shown on the left and the corresponding state-space model on the right. Purple ellipses correspond to protein expression, while the orange rectangles signify gene expression. All uppercase names are used for transcripts, and mixed upper-and lowercase is used for transcription factor names. A directed dashed line shows the direction of translation, while a directed solid line represents the direction of transcription regulation.

Figure 3 illustrates how tdGRN is used to model a multi-input network motif. In this example, the gene for the protein component of the yeast large (60S) ribosomal subunit, RPL16A, is transcriptionally regulated by three transcription factors: Fhl1, Rap1, and Yap5 (i.e., , ). Assuming that each TF has zero or some input delay to the regulation of RPL16A, the multiple time delay relationship can be described as follows:(5)

Recall that this *q × p* matrix captures the regulatory relationship between the  regulators and the  target gene.

The maximum number of input channels allowed in the model depends on the complexity of the motif structure and the time delay of each input channel. A greater number of available time points are required to model a more complicated network structure. Also, given a grossly limited number of time points, each additional unit of time delay reduces the number of available points to train a model and therefore reduces the reliability of the model. Consider an extreme case where a factor F regulates a gene G with 9 units of time delay. If there are only 10 time points, the regulatory relationship cannot be modeled since the data will show little or no evidence of regulation. In the case of Spellman's yeast microarray data (18 time points), tdGRN can compute a stable system for a maximum of four input and four input delays. In general, the maximum number of input channels is determined by trial and error and varies depending on the complexity of the network.

### 2.4. Network Connectivity

Rangel et al. [10] construct reliable gene regulatory networks based on bootstrap statistical analysis. The method is applied to highly replicated data. Their approach has a severe limitation, however, because most currently available time-course microarray data are either replicated few times (e.g., less than 5) or not replicated at all. Li et al. [14] use genome-wide location analysis results to construct a network structure and then infer the transcription factor activities with mathematical modeling. The latter approach significantly reduces the number of false positive node connections since the network connectivity is predetermined. In addition, the method can be used to model gene regulatory networks from nonreplicated data. The limitation of Li's approach is that it removes the power to uncover new connections that are not identified by ChIP-on-chip data.

In this paper, we present a three-step solution (tdGRN) such that network connectivity is based on, but not limited by, genome-wide location analysis results. First, the data is partitioned into two groups: transcription factors (TFs) and target genes (TGs). Each TF is a possible regulator of another TF and/or TG. Secondly, using the n4sid() function, tdGRN creates an initial set of network connections based on the location analysis results. All the TF versus TF and TF versus TG regulatory relations derived at this stage are screened for potential corresponding state-space models. Only the potential regulatory relations which satisfy the goodness-of-fit criteria are recorded and subjected to the next round of analysis. For each TF, tdGRN records the optimized parameters: initial state, number of time delays, the number of states (variables) that reflects the complexity of the regulations. In the third step, tdGRN performs an additional round of network connection screening based on the regulation parameters generated in the second step. For example, if a transcription factor F regulates *n* TGs with time delay , the tdGRN program will attempt to recruit other genes that have not been identified as targets of F but possess regulatory relations with F that resemble the existing ones. This is based on a common assumption that genes with high correlation in expression profiles are likely to be coregulated [1,2,22,23]. The additional round of network screening is implemented by MatLab's pem() function which is an alternative to the N4SID algorithm that uses a prediction error model (PEM) for parameterization. According to Favoreel et al. [24], the latter algorithm is relatively more sensitive compared to N4SID once the initial parameters are determined.

In addition, tdGRN generates a network output file that can be directly imported into Cytoscape [16] for network visualization, integration, and analysis.

## 3. Results

### 3.1. Data Sets

Two data sets are used in this study. First, an artificial data set is created to validate the model. There are several methods proposed in the literature to create appropriate artificial gene expression data [25,26]. The artificial data is created in this study by a method similar to that of Yeung et al. [26]; that is,  mimicking the periodic property of cell-cycle microarray data,  simulating the systematic errors in microarray experiments,  containing multiple time delay relations between regulators and targets. Secondly, we apply our model to analyze the yeast cell cycle microarray data published by Spellman et al. [21]. Details of both data sets are described in the following sections.

#### 3.1.1. Artificial Data

The artificial data consists of data streams of 2 regulators, R1 and R2, and 9 target genes, G1, G2,, G9. To simulate cell cycle gene expression data, the artificial data is created by using sine and cosine functions listed in Table 1. G1 to G3 are associated with R1 with delays  = 0, 1, 2, respectively. G4 to G6 are associated with R2 with delays  = 0, 1, 2, respectively. These relatively simple cases test the ability of the model to associate the target genes to their regulators, and to predict the number of the delays. G7 to G9 are associated with both R1 and R2 with delays  = 0, 1, 2, respectively. In these more complex cases, we test the ability of the model to connect the target genes to the multiple regulators, and to predict the number of the delays. Each data stream has a uniformly distributed random noise, , in the range of −0.05 to 0.05 (i.e., one twentieth of the range of sine and cosine functions), assigned to each time point.

**Table 1:** Parameters for the artificial data. The artificial data involves 2 regulators (R1, R2) and 9 genes (G1–G9).

Names	Function	Delay ()	Regulated by
Regulator R1		N/A	N/A
Regulator R2		N/A	N/A
Gene G1		0	R1
Gene G2		1	R1
Gene G3		2	R1
Gene G4		0	R2
Gene G5		1	R2
Gene G6		2	R2
Gene G7		0	R1+R2
Gene G8		1	R1+R2
Gene G9		2	R1+R2

#### 3.1.2. Yeast Cell-Cycle Data

The second data set used in this study consists of 800 expression profiles of alpha factor-based yeast cell-cycle genes studied by Spellman et al. [21]. The microarray hybridizations were done using asynchronous yeast cells sampled every 7 minutes for 18 time points. Normalized expression data were downloaded from the Stanford Microarray Database (SMD) [27]. No further pre-processing was done. The knnimpute() function from MATLAB's Bioinformatics toolbox was used to impute missing data.

In this study, it is assume that (1) the experimental time points capture biologically significant changes, but (2) there exist effects of hidden variables in the biological system that cannot be measured in a gene expression profiling experiment, for example, missing data for mRNA degradation.

In the following, we first describe the output of modeling the artificial data and the lessons learned in the modeling process. Then we present the results of modeling the yeast cell-cycle expression data. The global regulatory network diagram is presented as well as detailed analysis of G1- and B-type cyclins. Finally, we illustrate the capability of tdGRN in selecting the most feasible regulatory mechanism from multiple models.

### 3.2. Modeling a Gene Network Using the Artificial Data

**Figure 4:**
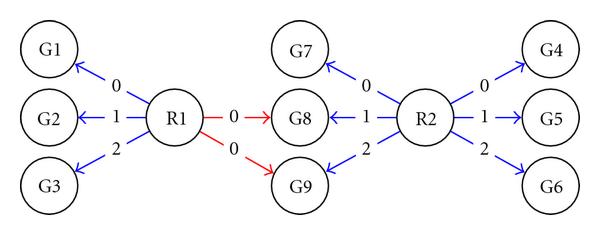
**SISO output for artificial data**. All edges are labeled with the predicted time delays. A blue edge represents a correct interaction; a red edge represents an incorrect one.

To demonstrate the difference between the SISO and MISO models, we first apply only SISO to network prediction on the artificial data. The two regulators, R1 and R2, are expected to connect to the target genes, G1 to G9, as described in Table 1. Figure 4 is a graphical representation of the produced SISO network. The network visualization is generated using Cytoscape where each node represents a gene and each directed edge represents a predicted regulatory relationship between a regulator and the target gene. Each edge is labelled with the predicted number of input time delays. Eleven out of twelve edges are identified by tdGRN-SISO. Among the eleven, 9 edges are annotated with the correct time delays. The complete output of tdGRN-SISO is tabulated in Table 2. The "Order" column gives the order of the system that reflects the model complexity. "Fitness (%)" (percentage of fitness) reflects the goodness-of-fit of the state-space model to the data. The "AIC" column contains the Akaike's Information Criterion score. The best-fitted model is selected by minimizing the AIC score. 

**Table 2:** SISO output for the artificial data.

Regulator	Target	Order	Delay ()	Fitness (%)	AIC
R1	G1	1	0	98.76	−9.0735
R1	G2	1	1	98.76	−9.1076
R1	G3	1	2	98.68	−8.9094
R1	G8	2	0	82.40	−3.9379
R1	G9	1	0	81.44	−3.2183
R2	G4	1	0	98.70	−8.8321
R2	G5	1	1	98.66	−8.9243
R2	G6	1	2	98.82	−9.1675
R2	G7	2	0	85.69	−5.0339
R2	G8	2	1	82.82	−4.3840
R2	G9	2	2	83.31	−4.2520

The results show that the SISO model can predict 100% correctly the one-to-one regulations but not the many-to-one regulations. For many-to-one regulations, the SISO model detects 5 out of 6 (83%) of them, but only 3 out of 6 are predicted with correct delays. As expected, almost all predicted connections (4 out of 5) from the many-to-one regulation are in higher-order state-space systems (i.e., second-order state-space systems) compared to the rest. tdGRN-SISO predicts a more complex regulation mechanism in these systems and produces poorer scores for the percent of fitness and AIC. The fact that the SISO model can identify most of the regulatory relations in our simulation suggests that, in the absence of a priori knowledge of the network structure, the single-input single-output model may be used to detect more complex network connections but the number of time delays and the order of the system may need to be reassessed using a MISO model.

**Figure 5:**
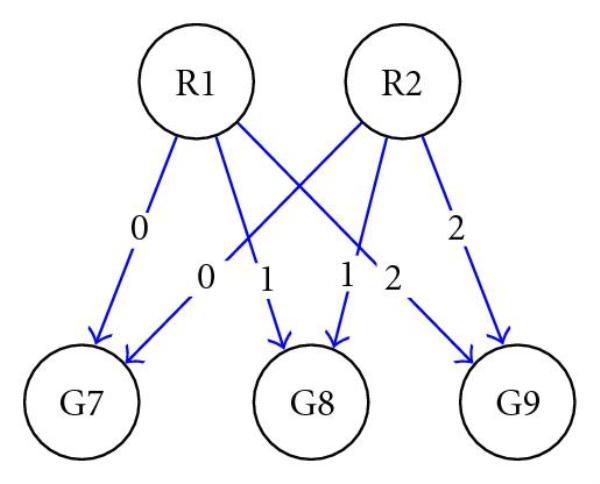
MISO output for artificial data.

We applied the tdGRN-MISO model for network prediction of the G7 to G9 genes. Given the knowledge that R1 and R2 co-regulate G7, G8, and G9, tdGRN-MISO can correctly predict 6 out of 6 edges and the corresponding number of time delays. Figure 5 is a graphical representation of the results. The complete output of tdGRN-MISO is shown in Table 3. Note that the tdGRN-MISO can produce much better models (better than 99% fitness, and much lower AIC scores) than tdGRN-SISO for these cases. The results illustrate the advantage of incorporating potential regulatory relationships into the modeling process.

**Table 3:** MISO output for artificial data.

Regulator	Target	Order	Delay (1,2)	Fitness (%)	AIC
R1,R2	G7	1	0,0	99.27	−8.0843
R1,R2	G8	1	1,1	99.18	−8.6052
R1,R2	G9	1	2,2	99.15	−8.4814

### 3.3. Modeling the Gene Networks in Saccharomyces cerevisiae

#### 3.3.1. Learning the Network Structure

The genome-wide location analysis results of nine known cell-cycle related transcription factors (Swi4, Swi6, Mbp1, Mcm1, Ace2, Swi5, Fkh1, Fkh2, and Ndd1) were from the study of Young's lab [28]. The results are reported as -values that reflect the significance of the binding between TFs and the corresponding promoter regions. We considered a -value less than or equal to 0.01 as being significant. This cutoff is less stringent than the 0.001 cutoff proposed by Lee et al. [20]. A relaxed threshold was selected to reduce the number of false negatives in location analysis. Complementarily, the number of false positives is controlled by providing cross-validation evidence from the modeling of time-series gene expression data. Based on the location analysis results and the selected cutoff, we identified 301 out of 800 cell-cycle regulated genes reported by Spellman et al. [21] which bound to at least one of the nine TFs. Refer to Table  1 in the supplementary material available on line at doi: 10.1155/2009/484601. for the list of the 301 genes and the binding map to the nine TFs. In that table, a "" character in a cell represents a significant binding ().

#### 3.3.2. Modeling the Gene Network

We applied tdGRN to the 301 cell-cycle regulated genes identified above. It predicted the regulation models of 93 genes or approximately 31% of the total input genes. The results are tabulated and shown in Supplementary Table  2. On a Pentium III 800 MHz computer, the total run time for tdGRN to analyze the 301 genes was approximately 90 minutes.

Almost half of the 93 genes are regulated in the G1 phase and about 25% are regulated in the G2/M phase. Compared to the 301 input genes, this represents a minor increase in percentage of genes regulated in G1 phase (36% to 44%), and a slight decrease for M/G1 phase (17% to 12%). The differential success rates in modeling G1- and M/G1-regulated genes may be due to the differences in the number of TFs from each phase. There was no M/G1-specific transcription factor used in this study. On the other hand, there were three (Swi4, Swi6, Mbp1) G1-activated TFs.

**Figure 6:**
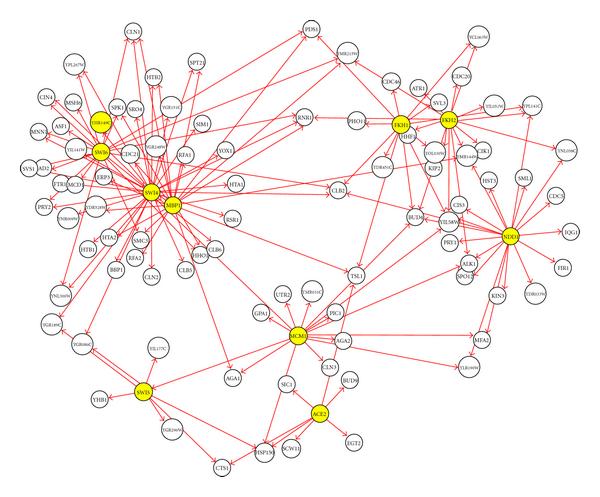
**Gene regulatory network of 93 cell-cycle regulated genes**. For greater clarity, genes are represented by white nodes and transcription factors are represented by yellow nodes. All node labels are shown using capital letters, irrespective of whether the node represents a transcription factor or a regulated gene. There is no significance to the size of circle used to represent nodes.

Among the nine transcription factors, Swi4, Swi6, and Mbp1 are known to play important roles in G1 and late G1 phase gene regulation [28,29]. The three TFs constitute two transcription factor complexes: SBF (Swi4 and Swi6), and MBF (Swi6 and Mbp1). SBF and MBF control over 50% of the total detected regulatory relations in our model. Figure 6 depicts the modelled network. In this network diagram, each yellow node represents a TF and each white node represents a target gene. A directed arrow between a TF and a target gene node represents a detected regulatory relation. Figure 6 reveals a large cluster of target genes regulated by combinations of SBF and MBF (left side of Figure 6). The fork-head transcription factors Fkh1 and Fkh2, and Ndd1 regulate a smaller cluster of G2/M-phase expressed genes on the right of the network diagram. Among the modelled genes in the two most abundant phases, the regulation of G1 phase's G1-cyclins (CLN1, CLN2, and CLN3) and G2/M phase's B-type cyclins (CLB2, CLB5, and CLB6) are identified. The modelled regulatory mechanisms of the cyclins were further investigated. The results are discussed in the following subsection.

#### 3.3.3. Regulation of G1- and B-Type Cyclins

**Figure 7:**
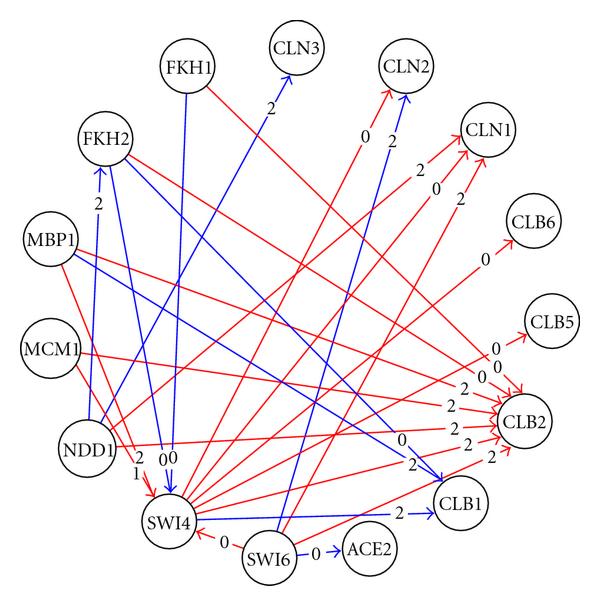
**Gene regulatory network for the G1- and G2/M-cyclins**. A red edge represents a known interaction based on location analysis and literature search; a blue edge represents an unknown relationship.

We examined more closely the regulation models of 3 G1-cyclins (CLN1, CLN2, and CLN3) and 5 B-type G2/M-cyclins (CLB1, CLB2, CLB4, CLB5, and CLB6). These two sets comprise all the CLN and CLB cyclins in the data set (CLB3 was not present). The CLN and CLB cyclins were selected due to their important roles in cell-cycle regulation and relatively well-studied regulatory mechanisms. Figure 7 is a diagram produced by tdGRN which features the selected genes. Each node represents a gene or a transcription factor, each directed edge represents a regulatory relation, and each edge label denotes the regulatory delay between two nodes. For example, Swi6→CLN2 has a delay of 2 samples (i.e., 2 × 7 minutes/sample = 14 minutes). The network edges are color coded such that a red edge represents a known interaction based on location analysis and a blue edge represents an unknown relationship.

The tdGRN technique uncovered a network of 15 nodes with 30 edges. 21 out of the 30 edges (i.e., 70%) have known regulatory relationships. The average model fitness is 67%. A tabulated output is provided in Table 4, in which the column "Order" means the order of the system which reflects the model complexity. The percentage of fitness reflects the goodness-of-fit of the state-space model to the data. AIC is the Akaike's Information Criterion score. Among the novel regulatory relations determined, there is evidence to support Swi6→CLN2 [29], Fkh2→CLB1 [30], Ndd1→FKH2 [31] regulation proposed in the literature.

**Table 4:** tdGRN output for yeast cyclins regulatory network.

Regulator	Target	Order	Delay ()	Fitness (%)	AIC	Binding evidence
Fkh1	CLB2	2	0	73.465076	−2.510095	Y
Fkh1	SWI4	2	0	71.941152	−3.016292	N
Fkh2	CLB2	2	0	69.618154	−2.677518	Y
Fkh2	SWI4	2	0	71.553277	−2.617587	N
Fkh2	CLB1	2	0	71.09556	−2.485664	N
Mbp1	SWI4	2	2	62.060827	−1.539307	Y
Mbp1	CLB2	2	2	64.177229	−2.604613	Y
Mbp1	CLN2	2	1	61.249674	−0.918211	Y
Mbp1	CLB1	2	2	60.996793	−2.303097	N
Mcm1	SWI4	2	1	67.744846	−2.430281	Y
Mcm1	CLB2	2	2	64.483538	−2.042422	Y
Ndd1	CLB2	2	2	73.178501	−1.628935	Y
Ndd1	CLN1	2	2	60.400341	−1.758735	Y
Ndd1	CLB6	2	0	71.960519	−1.687905	Y
Ndd1	CLB5	2	0	65.499866	−2.475658	Y
Ndd1	FKH2	2	2	72.445931	−3.800203	N
Ndd1	CLN3	2	2	65.957132	−2.655417	N
Swi4	CLB2	2	2	68.538231	−1.872905	Y
Swi4	CLN3	2	1	60.289651	−3.029056	Y
Swi4	CLN2	1	0	64.906209	−1.360941	Y
Swi4	CLN1	1	0	65.22902	−2.237727	Y
Swi4	CLB6	2	0	73.243562	−2.039319	Y
Swi4	CLB5	2	0	75.557682	−2.937196	Y
Swi4	FKH2	2	1	68.484476	−3.41278	N
Swi4	CLB1	2	2	77.002957	−2.119164	N
Swi6	SWI4	2	0	70.893228	−1.993188	Y
Swi6	CLB2	2	2	63.223062	−1.786548	Y
Swi6	CLN2	2	2	61.366645	−0.473000	Y
Swi6	CLN1	2	2	61.383819	−1.329583	Y
Swi6	ACE2	2	0	62.457434	−1.956277	N

#### 3.3.4. Regulation of CLN2

The tdGRN technique uncovered the regulatory relationship between Swi6 and CLN2 (with order = 2 and delay = 2) that is not reported in the location analysis results (see Supplementary Table  1). As mentioned in the previous section, Swi4 and Swi6 encode a heterodimer complex, SBF. It has been shown that SBF induces CLN2 transcription in the late G1 phase [28]. In our modeling, we detected the regulatory relations of Swi4→CLN2 with a first-order system (AIC score = −1.36), and Swi6→CLN2 with a second-order system (AIC score = −0.47). The difference in the AIC score indicates that although both TFs contribute to the regulation of CLN2, Swi4 represents a better model to control CLN2 regulation than Swi6. This finding is interesting in view of the observation that Swi4 is the DNA-binding component of the SBF complex and that interactions with Swi6 afford binding of Swi4 to DNA [31].

Using the SISO model, we demonstrated that Swi4 and Swi6 regulate CLN2 with input delays of 0 and 2, respectively. The fitness of the corresponding models is 65% and 61%, respectively. We applied tdGRN-MISO to this data in an attempt to improve the model of CLN2 gene expression. tdGRN-MISO produces 4 possible models (see Table 5). The best-fitted model based on AIC score (noted with an asterisk) is a first-order system with fitness equal to 67%, delays  and . Compared to the previously mentioned 2 SISO models, the MISO model is relatively better in terms of both AIC score and the overall percent fitness. These results suggest that Swi4 and Swi6 do regulate CLN2 transcription in a combined manner. This is in agreement with biological fact that Swi6 is the modifying factor whose translocation to the nucleus and binding to SWI4 are required for Swi4 to bind to DNA [32].

**Table 5:** MISO output for the CLN2 regulation.

Regulators (R1,R2) Target	Order	R1	R2	Fitness (%)	AIC	Best fit
		Delay	Delay			
(Swi4, Swi6) CLN2	1	0	1	64.798724	−2.566028	
	1	0	2	66.922105	−2.889358	
	2	0	0	67.240317	−0.892564	
	2	0	1	65.115198	−0.894492	

#### 3.3.5. Regulation of CLB2

CLB2 encodes a B-type cyclin that activates the cyclin-dependent kinase, CDC28, to promote the transition from G2 to M phase of the cell cycle. The promoter region of the CLB2 gene contains cis-element binding sites to 10 different transcription factors [33] according to Harbison et al. [34]. The binding motifs are also confirmed by the ChIP-on-chip results (see Supplementary Table  1). Using the cutoff of , seven out of nine TFs (i.e., Fkh1, Fkh2, Ndd1, Mcm1, Mbp1, Swi4, and Swi6) show significant in vivo binding to CLB2.

**Figure 8:**
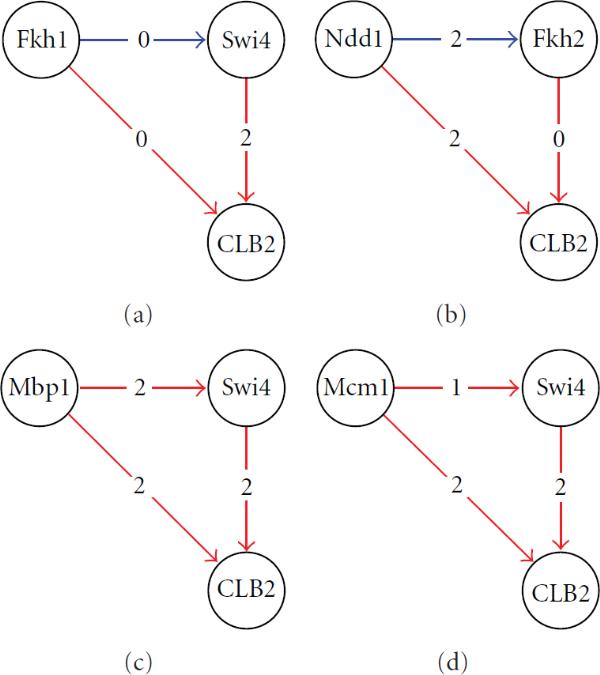
**Feed-forward loop network motifs in the regulation of CLB2 found by tdGRN**. Each edge is labeled with the value of time delay. A red edge represents a known interaction based on location analysis and literature search; a blue edge represents an unknown relationship.

The transcription factors that are found at the CLB2 promoter regions are known to regulate genes at different cell-cycle phases. For example, the SBF (Swi4, Swi6) and MBF (Swi6, Mbp1) complexes promote G1 to S phase transition, Mcm1 regulates late G2 and some M/G1 genes, and Ndd1 functions at the G2/M phase [30]. Hence, it is unlikely that all binding factors are functional and are active at the same time. Using the tdGRN, we detected regulatory relationships of the seven TFs to CLB2 (see Table 4). Furthermore, a closer look at the regulation of CLB2 reveals four feed-forward loop (FFL) network motifs (see Figure 8). A network motif is a biochemical wiring pattern that recurs throughout transcriptional networks. The feed-forward loop (FFL) is one of the most common network motifs found in the bacterium *Escherichia coli* and the yeast *Saccharomyces cerevisiae* [35]. A feed-forward loop is a three-gene motif incorporating two input transcription factors: a master and a secondary regulator. The master regulator regulates the secondary regulator and they both jointly regulate a target gene. We present the four FFLs found by the tdGRN-SISO in Figure 8. The top-left node is the master node of the FFLs. They are Fkh1, Ndd1, Mbp1, and Mcm1. The top-right node is the secondary regulator and this is Swi4 except when the master node is Ndd1 in which case the secondary regulator is Fkh2. The average SISO model fitness for each TF→CLB2 regulation is 68%. All TFs except the fork-head TFs, Fkh1 and Fkh2, have delay of 2 sampling intervals. Among the four FFLs, the regulatory relationship Mcm1+Swi4→CLB2 is also reported by Simon et al. [28] as an FFL using only the location analysis data with .

Mangan and Alon [35] suggest that one important function of FFLs is to speed up the response time of the transcription networks. That is, although positive gene regulation can be efficiently achieved by increasing the concentration of the TF gene's protein product, the response time is governed by the lifetime of the protein product, which is often much longer. Therefore, one way to speed up the response is to increase the degradation rate of the protein product through a second regulator and perhaps to block access to the target gene's binding site by the first TF protein product. Since the later regulator controls the expression of the former TF (the secondary regulator) and the target gene, it is called the master regulator. At the transcript level, one would expect the target gene expression level to be a function of the expression of both regulators as the FFL mechanism should be functional.

We applied tdGRN-MISO to the four FFL motifs identified by the SISO model for CLB2 regulation. We hypothesize that if an FFL is present, one would expect the master and secondary regulators to work in a collaborative manner. That is, the unexplained variation seen in the principal TF's regulation can be elucidated by the feed-forward regulation of the secondary TF, and vice versa. On the other hand, if the FFL is inactive or if only one of the two regulators works, then the model will not be improved by tdRGN-MISO and the percent fitness of the model will remain roughly the same or be worse.

The output of tdRGN-MISO is tabulated in Table 6. The best-fitting model is marked with an asterisk in the rightmost column. The best model for Fkh1+ Swi4→CLB2 at 80% fitness is a first-order system with zero time delay for Fkh1 and 2 time delays for Swi4. The best model for Mbp1+Swi4→CLB2 is a second-order system with zero time delay for Mbp1 and Mbp2 time delays for Swi4. The fitness is ~82%. We did not observe significant improvements in terms of percent fitness for the Ndd1 + Fkh2 and Mcm1 + Swi4 models. This suggests that only the former two out of the four possible FFLs are likely to control CLB2 regulation, and indicates improvements in model fitness for the Mbp1 + Swi4 and Fkh1 + Swi4 models over the Ndd1 + Fkh2 and Mcm1 + Swi4 models, which supports our hypothesis.

**Table 6:** Multiple-input and single-output regulatory relations for CLB2.

Regulators (R1,R2) Target	Order	R1	R2	Fitness (%)	AIC	Best fit
		Delay	Delay			
(Fkh1,Swi4) CLB2	1	0	1	63.644575	−1.537761	
	1	0	2	79.773793	−3.229595	
	1	1	0	61.814689	−1.510308	
	1	1	2	70.844626	−1.853543	
	2	0	0	71.974595	−1.759781	
	2	0	1	76.75773	−2.140303	
	2	0	2	75.445502	−2.533515	
	2	1	0	74.935231	−3.046253	
	2	1	1	74.816372	−1.912746	

(Ndd1,Fkh2) CLB2	1	0	1	61.692141	−0.711095	
	1	0	2	69.823501	−2.268478	
	1	1	0	60.652689	−0.637223	
	1	1	2	74.517609	−1.745955	
	1	2	0	72.64509	−1.793645	
	1	2	1	69.148994	−1.644347	
	2	0	0	74.392399	−0.98651	
	2	0	1	75.908715	−1.714605	
	2	0	2	78.764701	−1.941071	
	2	1	0	71.297965	−0.877083	
	2	1	1	74.106334	−1.081118	
	2	1	2	69.865951	−2.514543	
	2	2	0	70.654615	−1.453532	
	2	2	1	70.375696	−2.480809	

(Mbp1,Swi4) CLB2	1	1	0	65.281567	−1.531062	
	2	0	2	82.284591	−2.228897	

(Mcm1,Swi4) CLB2	2	0	0	76.835382	-1.577900	
	2	0	1	78.146638	−1.707728	
	2	1	1	76.606035	−1.755346	
	2	1	2	60.319422	−1.722575	
	2	2	0	63.90925	−1.804213	
	2	2	1	65.281011	−1.704606	
	2	2	2	60.778085	−2.139843	

## 4. Discussion

Transcription is a very complex process that entails assembly of multiprotein complexes and enzymatic reactions, and the ultimate transcript output also depends on temporal factors that are not amenable to accurate analysis. In gene-gene interactions and in multigenic interactions (networks), the temporal aspects have appreciable biological consequences but these causal factors not readily deciphered. Delineating all these in terms of reverse engineering a genetic system requires collections of large and replicated data points that are commensurate with the complexity of the system and its components and also requires the computational power to analyze the data. Both can present difficulties, considering the inherent complexity. Against this backdrop is the adaptation of models that have originally been used in reverse engineering physical systems. State-space model is one such method. It has the advantage of taking the dynamic changes of gene expression into consideration unlike static models such as hierarchical or -means clustering. The data points (gene expression levels) are treated as observation variables that arise from linear combinations of the internal variables in the living system that are intractable due to technicality or impracticality. This method is adaptable to data collection that is missing some points and also to data that are not highly replicated.

Transcriptomics studies have generated the most extensive datasets in genomics. Microarray analysis is being used increasingly to determine the expression patterns of tens of thousands of genes simultaneously. When the expression pattern of the same genes under two or more intracellular conditions (e.g., due to innate physiological changes or due to changes in the growth temperature) is determined, there is a potential opportunity to discern gene-gene connectivity with respect to the changing internal environment. However, microarray data only measures the steady-state levels of the RNA product, and all other factors such as the level of DNA-binding factors (e.g., transcription factors; TF) are hidden variables. A pertinent question here is, "what is the impact of the delay in making the product of Gene 1 on Gene 2 if Gene 1 is impacting the transcription of Gene 2?" In this regard, the model developed in this study is useful.

### 4.1. Discrete versus Quantitative Models

Yeast cell-cycle regulated genes demonstrate a periodic pattern [20]. The gene expressions are known to be phase specific. The expression data are reported as (*sample expression/reference expression*). That is, one measures the changes in expression with respect to a common reference instead of absolute expression. A 2-fold change in expression, that is,  1, is generally considered significant. It is important to note that a negative  does not imply inactivity of a regulator. Instead, it means that the gene expression level is relatively lower (by the fold change) compared to the control sample, for example, the time zero sample.

Among the five state-space or Bayesian network solutions referred to in this work, the models published by Sung et al. [13] and Li et al. [14] use discrete (binary or Boolean) profiles of gene expression. However, discrete models suffer from an inherent difficulty. Finding a reasonable threshold to define the inactive and active states of gene expression is a nontrivial task. The basal level of expression varies by several orders of magnitude among some genes. In such cases, the fold-change values alone cannot define the on-off state. For a gene whose state is defined as "off" in a discrete model because of a fold-decrease value at time = 1 might, in fact, still be substantially active if its basal level was high (at time = 0). Consequently, the on-off states of various genes in a microarray are not definable on the basis of comparing their fold-changes alone. Soinov et al. [36] have proposed an alternative method to bypass the assumption of arbitrary discretization thresholds for the regulators. Their states of a "predicted gene" (i.e., a target gene) are determined by the quantitative expression levels (or changes in the expression with respect to a control sample) of the "explaining genes" (i.e., the regulators). The results are presented in the form of a rooted decision tree such that the states (up-/down-regulated, or expressed/not expressed) of a target gene (leaf node) are determined by the combinatorial decision rules of the regulators (nonleaf nodes). The Soinov et al. approach [36] can potentially improve the performance of discrete network models.

On the other hand, the biggest challenge in quantitative modeling is the inherent noise in the expression data. Especially when a gene is expressed at a low level, a low signal-to-noise ratio causes an inaccurate measurement of fold-change. This will in turn affect the ability of quantitative models in learning the network structure and in getting good model fitness. In this study, the average model fitness for yeast expression data is 67%.

### 4.2. Gene Regulatory Network: What, When, and How

A ChIP-on-chip experiment, in the context of our work, answers the question: what are the potential targets of a given TF? The evidence of in vivo protein-DNA interactions can help biologists to uncover regulatory network structure [14,20,28]. However, the binding of a protein to a gene sequence does not necessarily indicate a regulatory outcome. In yeast, a B-type cyclin, CLB2, is known to have cis-element binding sites for 10 different transcription factors [34]. Many of these transcription factors are known to regulate genes at different cell-cycle phases. It is unlikely that all binding factors are functional at the same time. Our modeling tool provides a way to model gene regulation based on time-course expression data. In this document, we analyzed 301 cell-cycle regulated genes with possible regulatory relationships to at least one of the nine known transcription factors. Among these, we are able to identify and model the regulation mechanisms of 93 (31%) genes.

Analysis of the time taken by a gene to reach its full expression level (peak time) provides insights into when a gene is maximally expressed during the cell cycle. The understanding of gene expression timelines is useful for associating a time factor to the physiological changes in cells. However, the duration for a gene to reach its peak expression in a cell-cycle alone is not enough to constitute the full picture for gene regulation. For example, the transcription factor complex, SBF (Swi4 + Swi6), regulates CLN1 and CLN2 transcription in the late G1 phase and drives the transition into S phase. The peak times for Swi4 and Swi6 are 13% and 37%, respectively. The peak times for the SBF regulated genes CLN1 and CLN2 are 25% and 23%, respectively. One component of the SBF regulator, Swi6, reaches the peak time later than both CLN1 and CLN2. This shows that the peak time analysis does not convey information on how genes are regulated. One may hypothesize that Swi4 is the rate determining factor in the regulation of the cyclins and that the G1 cyclins will quickly reach their peak expressions at 25% after Swi4 reaches its peak at 13%. Our modeling results support the above mentioned assumption (refer to Section 3). The Swi4 and Swi6 transcription factors regulate CLN2 transcription in a combinatorial manner. The percent fitness of the Swi4+Swi6→CLN2 model is better than two separated single-input and single-output models. Interestingly, our modeling results also suggest that CLN2 is regulated by both Swi4 and Swi6, and CLN1 is regulated only by Swi4. This could be the result of relatively weaker role of Swi6 in cyclin regulation as Partridge et al. have shown that MCB core elements of both CLN1 and CLN2 depend primarily on SWI4 [37].

### 4.3. Model Overfitting

The tdGRN uses location analysis results to help identify the TF and target gene pairs. This significantly reduces the risk of overfitting by filtering out the unrelated inputs (i.e., unwanted noise). In addition, Akaike's Information Criterion [17] is applied to the model selection process. The AIC discourages the selection of a higher-order system by imposing a penalty for the complexity of the estimated model. It attempts to find the best goodness-of-fit with a minimum system complexity. This provides another guard against overfitting.

## 5. Conclusions and Future Work

We have developed a new modeling tool, tdGRN, for determining prospective gene regulation models from time-series gene-expression data. The tool has been demonstrated on artificial data and yeast cell-cycle gene-expression data. Using the yeast microarray data, we have illustrated that our model can help identify regulatory relations with multiple time delays. The model complements ChIP-on-chip results by predicting the most probable gene regulatory relatioships between transcription factors and their target genes. The tool also identifies previously unknown regulatory relationships. For example, in the regulation of G1- and B-type cyclins, tdGRN uncovers 30 regulation relationships in a network with 15 nodes, 9 of which are novel findings. The existing literature contains support of these novel findings [29–31].

The tdGRN tool uses genome-wide location analysis data to reveal the primary network structure. Additional regulatory relationships can be determined by goodness-of-fit of alternate models. It should be interesting to compare this method to the learning-by-modification method developed by Sung et al. [13] where the network structure is based on a backward elimination mechanism. Another important facet of future work would be a systematic study of the effect of noise on tdGRN. The current version of tdGRN has a command line user interface. Some features can be implemented to increase user friendliness. Examples include a GUI and a facility to load multiple experiments.
